# Twenty-first-Century Skills: Teaching Empathy to Health Professions Students

**DOI:** 10.7759/cureus.36076

**Published:** 2023-03-13

**Authors:** Eva Peisachovich, Megha Kapoor, Celina Da Silva, Zipora Rahmanov

**Affiliations:** 1 Medical Education and Simulation, York University, Toronto, CAN; 2 Nursing, York University, Toronto, CAN

**Keywords:** skills, higher education, simulation, education, empathy

## Abstract

A key component of therapeutic relationships is the ability of medical professionals to empathize with patients, as research indicates a link between a healthcare worker’s ability to empathize with patients and improved patient outcomes. Empathy - the ability to perceive the meaning and feelings of another and to communicate those feelings to others - may be an innate concept, but it is shaped through behaviours and experiences. It is imperative, then, that post-secondary students entering the medical field be taught to develop empathy in order to facilitate positive patient outcomes. Embedding empathy-based education in the curriculum of medical, nursing, and allied health programs early in the course of study can help students understand the patient’s perspective and facilitate positive therapeutic relationships early in students' professional careers. The shift from traditional teaching and learning styles to online learning has created deficiencies such as gaps in communication, empathy, and the development of emotional intelligence. To address these gaps, new and innovative ways to teach empathy, such as simulation, can be employed.

## Editorial

Introduction

Healthcare largely focuses on providing care and support to patients who are at their most vulnerable. The development of relationships between medical professionals and patients is key to providing exceptional care. One key component of these relationships is the ability to empathize with patients [1]. Empathy is the ability to perceive the meaning and feelings of another and to communicate those feelings to others. It is essential, then, to provide students at the post-secondary level with opportunities to develop empathy, as this will allow students entering the healthcare field to understand the patient’s perspective. Patient care consists of a multidisciplinary team approach; hence the relationships between physicians, nurses, allied-health professionals, and the patient are integral to patient care outcomes.

Current research indicates that there has been a lack of empathy from medical professionals when caring for patients [1], which highlights the need for students to learn how to respond empathetically to situations and patients. Empathy-based education can provide a way for students to develop empathetic responses and behaviours [1]. Embedding empathy-based education in the curriculum of medical, nursing, and allied-health programs earlier in students’ course of study can prove beneficial for both students entering the healthcare field and their future patients.

Significance and social impact

While the widescale adoption of information technologies has catapulted production and progress in many areas of society, including innovation in education, it has left in its wake some deficiencies, like gaps in communication, empathy, and the development of emotional intelligence [1]. Not only are these skills necessary for a healthy society, but they also are a key component of success for our graduates, particularly those on interpersonal career paths such as nursing and social work. Notably, research has found that high emotional intelligence contributes to self-confidence, control, flexibility, and empathy and can be a better predictor of student success post-graduation than Grade Point Average [2].

Human capabilities and skills such as persuasion, social understanding, and empathy will become more and more prized over the next decade; these will become differentiators as artificial intelligence, machine learning, and automation take over our other tasks. Unfortunately, these human-oriented skills are often not recognized as primary skills. It is important to recognize strengths and weaknesses when it comes to emotional intelligence and to invest in developing and integrating mediums to both teach and embed emotional intelligence into the curriculum.

Over the years, students enrolled in medical education have been taught to remain detached from their patients to avoid becoming too involved in their patients’ care [3]. This notion of distancing oneself from one’s patients has been the focus of medical education since the 1960s [3] and is intended to prevent the emotional burnout and anxieties that may arise when working with patients who are ill. Yet research shows that a healthcare worker’s inability to empathize with patients has a negative impact on the goals of care for patients [4]. A study of patients with the common cold indicates that patients adhere better to treatment regimens when they perceive that their healthcare providers (HCPs) display empathy during their interactions [4]. Moreover, empathetic encounters between HCPs and patients have been found to result in positive relationships that can benefit patients by decreasing their levels of depression, anxiety, and distress, and increasing their levels of emotional well-being, satisfaction, and adherence to treatment regimens [4]. Thus, an empathetic HCP can positively impact patient outcomes. Further, a patient’s perceptions of an HCP’s level of empathy have been shown to affect the patient’s overall health outcomes [4]; patients who perceive their HCPs to be empathetic in their encounters experience better recovery from illness than those who feel their HCPs are not empathetic. Similarly, a retrospective analysis of psychiatrists who treat patients for depression shows that those practitioners who create a bond with their patients have better results in treating depression with a placebo than those who use active drugs but do not create a bond [4]. To date, no studies were found to address the optimal amount of empathy required to support these findings. In sum, empathy allows for a fuller understanding during a patient encounter and creates the basis for the therapeutic relationship going forward. These studies underscore the need to shift away from a traditional teaching approach that encourages detachment and toward empathy-based education that enables better relationships between HCPs and their patients and within multidisciplinary contexts. In addition to teaching students to understand their patient’s emotions in order to provide the best possible care, a curriculum or education program that encompasses empathy is intended to also teach students to manage their own emotions in order to mitigate anxiety and burnout.

Ways empathy is embedded into educational programs and curricula

Current programs for medical, nursing, and allied-health studies focus their curricula on providing direct patient care. Courses that focus on empathy or empathetic behaviours are provided as elective options for students. Currently, limited research has been conducted on the ways empathy-based education is embedded into educational programs [1]. One group of researchers created a caring professionals program to foster and facilitate empathy and empathetic behaviours [1]; it can be embedded into current curricula of medical, nursing, and allied-health programs. Such empathy-focused education initiatives support a shift away from mainly didactic educational methods and towards more innovative methods that allow students to experience and express themselves. A key element of empathy-based education is a hands-on experience, which includes the use of simulation-based approaches, reflection, problem-solving, didactic lectures, active participation, and role modelling. While nursing programs do focus on the six elements of empathy-based education, current research suggests that as students in both medical and nursing programs progress into their third and fourth years of education, levels of empathy decrease [1,3]. Possible explanations for this shift in empathetic response levels include increases in time pressures, amounts of material to learn, use of technology, and patient interactions; the rise of evidence-based practices; and the lack of role models [3]. Most empathy-based research focuses on quantitative research studies that highlight empathy as a measurable factor in patient care. There is limited research on qualitative studies on empathy that highlights the perspectives of patients, physicians, nurses, and students in medical, nursing, and allied-health programs. Other available research includes literature reviews that highlight simulation-based education-role playing, simulated-person methodology, serious games, and virtual reality as beneficial means of teaching empathy in medical, nursing, and allied-health programs [5]. The use of simulation-based education in these programs assists students in learning the communication skills needed to support empathetic responses and behaviours.

A study highlighted in one such literature review shows that the use of role-play and a debriefing process results in a significant improvement in the participants’ understanding of the patient’s perspective [5]. This review further indicates that the use of simulation in education may combat the decline of empathy reported during the later years of study for nursing and medical students as it allows students to manage both a less idealistic view of healthcare practice and an appropriate level of identification with patients [5]. While these studies suggest promising results, more research on empathy-based education in allied-health programs is needed.

Education has been changing in various ways over the past few years, such as the shift to a more digital teaching environment. Further, new and innovative ways of teaching empathy are being employed at the post-secondary level. One method of teaching empathy that has gained popularity is a simulation, which employs simulated persons, case studies, role-playing, games, and virtual/augmented reality. Given the established link between the empathy of HCPs and improved patient outcomes, it is critical to embed methods that afford healthcare students an opportunity to access a safe space in which to practice reacting empathetically, being self-aware, and learning from mistakes. While simulated methods of teaching may not entirely replicate a real-life experience that a student would have in the field, simulated techniques allow students to develop the building blocks with which to shape the empathetic behaviours and responses that they can use in practice.

Transforming health professions: humanizing education by using simulation methods

What humans have to offer - what humans can do better than any smart machine - is related to the people around them. This skill needs to be both recognized by and embedded into the education context; we must invest in emotional intelligence in the same way we invest in the more technical parts of our careers.

Over the years, empathy has been taught as a skill in medical and nursing education. Conversely, medical students have also been taught how not to become too attached to patients when providing care. The fact that students can be taught (or taught to avoid) empathetic behaviours highlights that empathy is a social construct that is developed through interactions yet is also a natural and innate concept. Thus, a social-constructivist approach to learning or teaching empathy focuses on human interactions and experiences. While research suggests that medical students’ empathy levels start off high but decrease by their third year of medical school [3], the converse is true for nursing students; their empathy levels start off low and increase as the program continues. Research also highlights disparity in empathy levels between genders, with women being more empathetic than men [3]. Levels of empathy also vary according to professional roles; roles with less direct patient contact correspond with lower levels of empathetic skills [3]. To address these gaps and enable students to develop this skill, empathy should be taught in all years of programs in healthcare fields. This shift is needed to continue to facilitate positive patient outcomes and to build strong therapeutic relationships between patients and their healthcare professionals.

Conclusion

Empathy is an innate concept that is shaped through behaviours and responses. Patient care requires empathy to facilitate a positive therapeutic relationship. Showcasing empathetic behaviours and responses during direct patient care leads to positive outcomes for patients in relation to their health concerns and diagnoses. To fully access these benefits, a shift from didactic educational methods to experience-based learning is needed in order to enable students to learn how to use empathetic behaviours and responses in their future practice. The shift to a more digital world has opened doors for creative and innovative methods, such as simulation, to teach empathy. Simulation provides a safe space for students to make mistakes, and gain valuable feedback, thus enabling them to foreground empathy in their future practice. Current medical, nursing, and allied-health programs do not need to revamp their entire curriculums to facilitate this shift; minor adjustments can be made to include experiential learning that includes reflection, role-playing, problem-solving, active participation, and role modelling as ways to develop empathy.
